# Maternal and neonatal factors’ effects on wharton's jelly mesenchymal stem cell yield

**DOI:** 10.1038/s41598-024-72386-z

**Published:** 2024-10-17

**Authors:** Ranim Mahmoud, Mohamed Bassiouny, Ahmed Badawy, Ahmad Darwish, Sohier Yahia, Nora El-Tantawy

**Affiliations:** 1 Pediatric Department, Faculty of Medicine, Mansoura University, Mansoura, Egypt; 2 Obstetric and Gynecology Department, Faculty of Medicine, Mansoura University, Mansoura, Egypt; 3 Mansoura Research Center for Cord Stem Cells (MARC-CSC), Faculty of Medicine, Mansoura University, Mansoura, Egypt; 4 Medical Parasitology Department, Faculty of Medicine, Mansoura University, Mansoura, Egypt

**Keywords:** Wharton's jelly, Stem cells, Yield, Maternal, Neonatal, Flow cytometry, Stem cells, Medical research

## Abstract

As Wharton's jelly-derived mesenchymal stem cells (WJ-MSCs) are easily accessible, easy to isolate, and ethically acceptable, they represent a promising source of MSCs for use in regenerative medicine. Considering decisions on WJ-MSCs collection requires extensive knowledge of the factors that impact their yield. This study's aim was to evaluate the influence of parameters related to mothers and newborns on the WJ-MSCs yield. The WJ-MSCs were isolated and expanded after being isolated from 79 umbilical cord (UC) samples. Population doubling time and cell proliferation were assessed. By flow cytometry analysis, WJ-MSCs were identified by positivity of CD105, CD90, and CD73 and negativity of CD45 and CD34. There was a statistically significant negative correlation between UC width and P1 doubling time. Maternal age and WJ-MSC yield were shown to be negatively correlated. Birth weight and gestational age showed a significant positive correlation between WJ-MSCs yield and neonatal variables. No significant correlations were detected between the WJ-MSCs and the mother parity, nor the neonatal sex, fetal presentation, or head circumference. The WJ-MSCs yield increases with younger maternal age, higher gestational age, and increased neonatal birth weight. Hence, consideration should be given to these factors when selecting the ideal donors.

## Introduction

The stem cells are a special type of cells that have a potentiality for regeneration and thus can maintain and repair cellular damage. So, it have a promising therapeutic prospective in cell transplantation. Mesenchymal stem cells (MSCs), hematopoietic stem cells, and other progenitors are among the cells from different origins that have been isolated^1^^,^^2^. Mesenchymal stem cells (MSCs) isolated from bone marrow have been the focus of most studies; however, MSCs may also be obtained from amniotic fluid and UC blood^3^ and chorionic villi^4^. The mesenchymal stem cells have been also derived from the Wharton's jelly, the connective tissue that envelops the two arteries and vein in the UC^5^.

WJ-MSCs have grown increasingly common in recent years due to their inherent advantages than MSCs obtained from other sources. These advantages include improved ex-vivo growth capacities, rapid proliferation, fewer graft-versus-host complications, and a reduced risk of teratomas^6^. In addition, compared to bone marrow and embryonic stem cells, which are frequently thrown out as human waste, there are no ethical concerns regarding their acquisition^7^. Wharton's jelly mesenchymal stem cells (WJ-MSCs) possess paracrine activity, which is the ability to alter their environment by releasing bioactive chemicals and compounds known as secretome. This activity is the primary reason for the therapeutic potential of WJ-MSCs. The secretome has anti-inflammatory and anti-fibrotic properties, but it also carries out several biological activities like immuno-modulation, tissue replenishment, and cellular homeostasis^8^. Previous researches recommended its use for cancer cells growth inhibition^9^, ameliorating diabetic nephropathy and hepatopathy complications as a novel protective approach^10^, the reduction of the severity of the inflammation and immune response against Covid-19 because of its anti-inflammatory and immunomodulatory characteristics^11^, and reduce fibrosis caused by bleomycin-induced lung injury^12^.

There are many approaches to identifying mesenchymal stem cells as their capacity to adhere to flexible surfaces and their spindle-like appearance in culture and they can differentiated into adipocytes, chondrocytes and osteocytes. Furthermore, WJ-MSCs should express CD105, CD73 and CD90 and not display CD34, CD45, CD19 or CD11b, CD79 alpha or CD14, or HLA-DR surface molecules, according to the International Society for Cellular Therapy (ISCT)^13^. The amount of obtained stem cells is insufficient because isolating cells frequently involves an invasive and technically difficult process that compromises the quality and biological activity of the obtained cells and hinders their capacity to multiply and differentiate during in vitro culture^14^. It is essential to gain a good yield from the WJ-MSCs. Many factors have been suggested to impact the quantity of WJ-MSCs isolated from Wharton’s jelly, and which may be responsible for the variations in the reported results. These variables include the mother's age, the gestational age, mother’s parity and gravidity, the newborn's sex, and the birth weight, all of which might affect the amount of CD34 + cells in the blood^15^.

Considering the maternal and neonatal factors in the selection of promising donors for isolation of a good yield from WJ-MSCs, our study aimed to determine whether maternal parameters-such as the mother's age, gravidity, parity, and other health-related conditions—and neonatal parameters—such as birth weight, sex, head circumference, and UC width—may affect the yield of WJ-MSCs.

## Patients and methods

The Institutional Review Board Mansoura Faculty of Medicine, Mansoura University, Egypt approved the current study, and informed consents were obtained from all participants before delivery. A total of 100 pregnant women were included in the study. Women with temperature higher than 38 °C, having premature rupture of membranes, with multiple gestation, positive for blood transmitted infections or with proved fetal malformations were excluded from the study. The patient records were reviewed for data on pregnancy history, number of prior pregnancies, parities, the gestational age, newborn sex, newborn weight, and head circumference, the width of the UC, gestational diabetes, pre-eclampsia, and other reports of maternal co-morbidity. Patients' records and gathered data for the study were kept confidential and experiments were performed in accordance with relevant guidelines and regulations.

### Samples collection and cord processing

Following cesarean birth, a total of 100 fresh human UC samples were aseptically collected from the middle segment. A collection cup holding in Dullbecco's Modified Eagle Media (DMEM) with 4500 mg/mL glucose and antibiotic solution (0.2% streptomycin, 0.12% penicillin and 0.1% gentamicin) (Lonza, Belgium) was used to hold the roughly 5 gm of UC that were aseptically collected. The samples were subsequently kept at 4 °C. All samples were processed within 2–4 h of collection. After that, the samples were sent to the Mansoura University Stem Cell Research Center lab to be processed. In order to remove blood clots, samples were processed in a biosafety cabinet and washed using ice-cold phosphate buffered saline repeatedly.

**Fig. 1:**
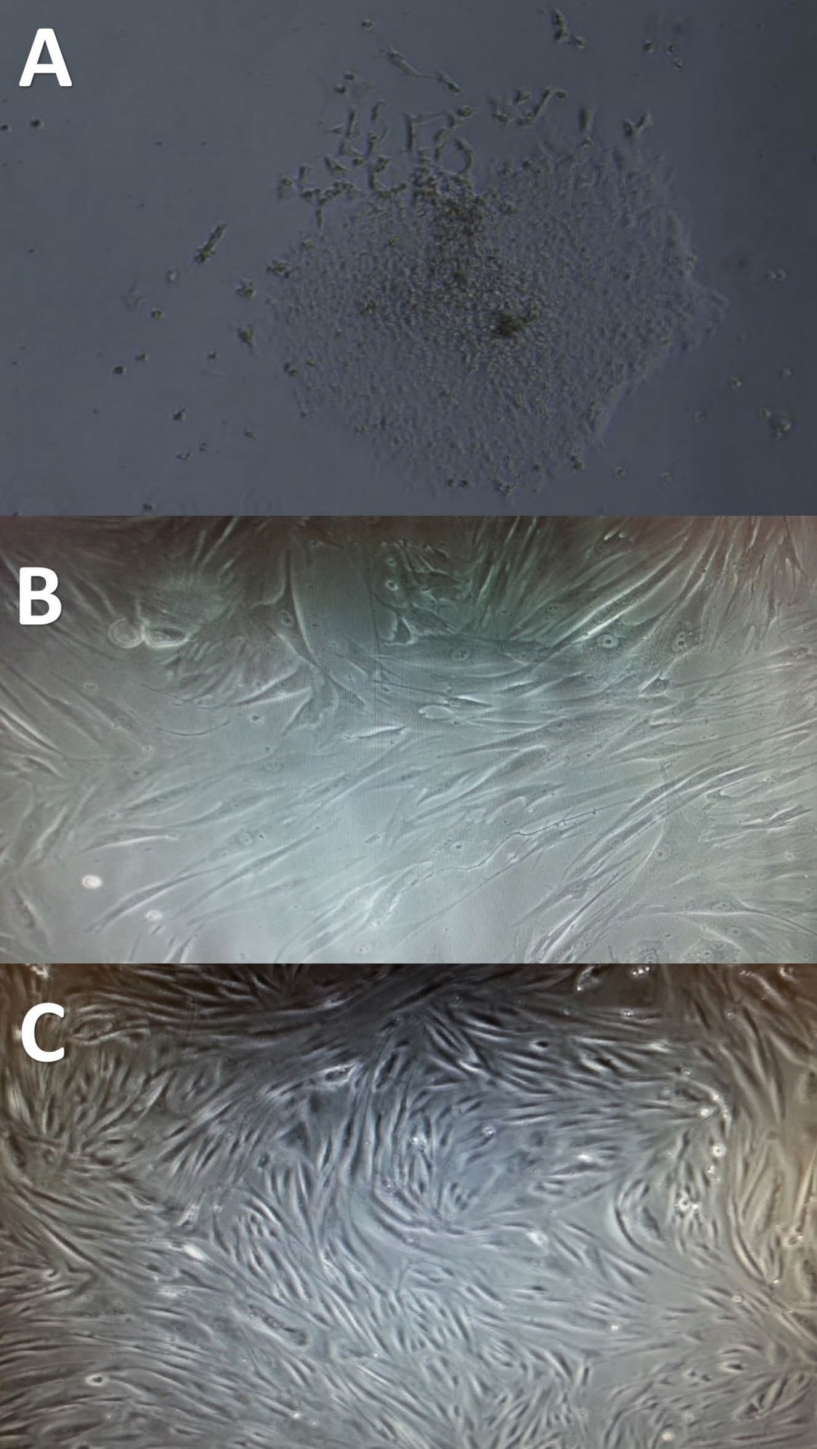
Wharton’s jelly mesenchymal cultured cells showing (**A**) Initial outgrowth of spindle shaped cells after 10 days in culture, (**B**) Wharton’s jelly mesenchymal cells with 50% confluency, (**C**) homogeneous population of fibroblast-like MSCs with 90–100% confluency.

The explant method was utilized to isolate mesenchymal stem cells from the UC Wharton's jelly^16^. Six to nine pieces of the explant outgrowth are cut off and put on the culture dishes, where they remain until the jelly solidifies. Next, a culture media is included. The Dulbecco's Modified Eagle's Medium (DMEM) contains 4 mM L-glutamine, 4500 mg/L glucose, 1 mM sodium pyruvate, and 1500 mg/L sodium bicarbonate. The culture dishes are kept for 3–4 days at 37 °C in a humidified atmosphere with 5% CO_2_. Following the attachment of Wharton jelly, the dish media is replaced every 2–3 days, and after about 7–10 days, the cells displayed the WJ-MSCs phenotype will be recovered. The cells were separated by the use of 1–2 mL of commercial trypsin solution in a 25 cm^2^ culture flask before being incubated for 3 min. at 37 °C. Using a tissue culture centrifuge, the mixture was centrifuged at 1500 × g for 3 min to neutralize the trypsin. One to two milliliters of culture medium were added. To carry out amplification and characterization, the pelleted cells—which are regarded as passage 0 (P0)—were re-suspended in a culture medium, counted, and sub-cultured at a 1 × 10^4^/cm^2^ seeding density (Fig. 1).

### Cell viability determination and population doubling time determination

The cell viability was determined using the Trypan blue test as described by^17^. After the isolated cells were stained with Trypan blue, the number of viable (white) and non-viable (blue) cells was determined using a hemocytometer composed of nine 1 × 1 mm (1mm^2^) squares and a microscope (AX-71, Olympus Corporation, Shinjuku-ku, Japan). Every experiment was carried out three times in duplicate.

Trypsin was used to harvest the cells at 80–90% confluence after each passage. The cells were then plated in a T25 cell culture flask, counted, and re-plated till the third passage. The population doubling time (PDT) was calculated according to ^18^. The following formula was used for calculating PDT, which was expressed in hours: PDT is equal to (lgNt – lgN0)/lg2, where t is the culture period, Nt is the harvested cell count after the cell passage, and N0 is the number of cells seeded at the start of the passage.

### Flow cytometry

**Fig. 2:**
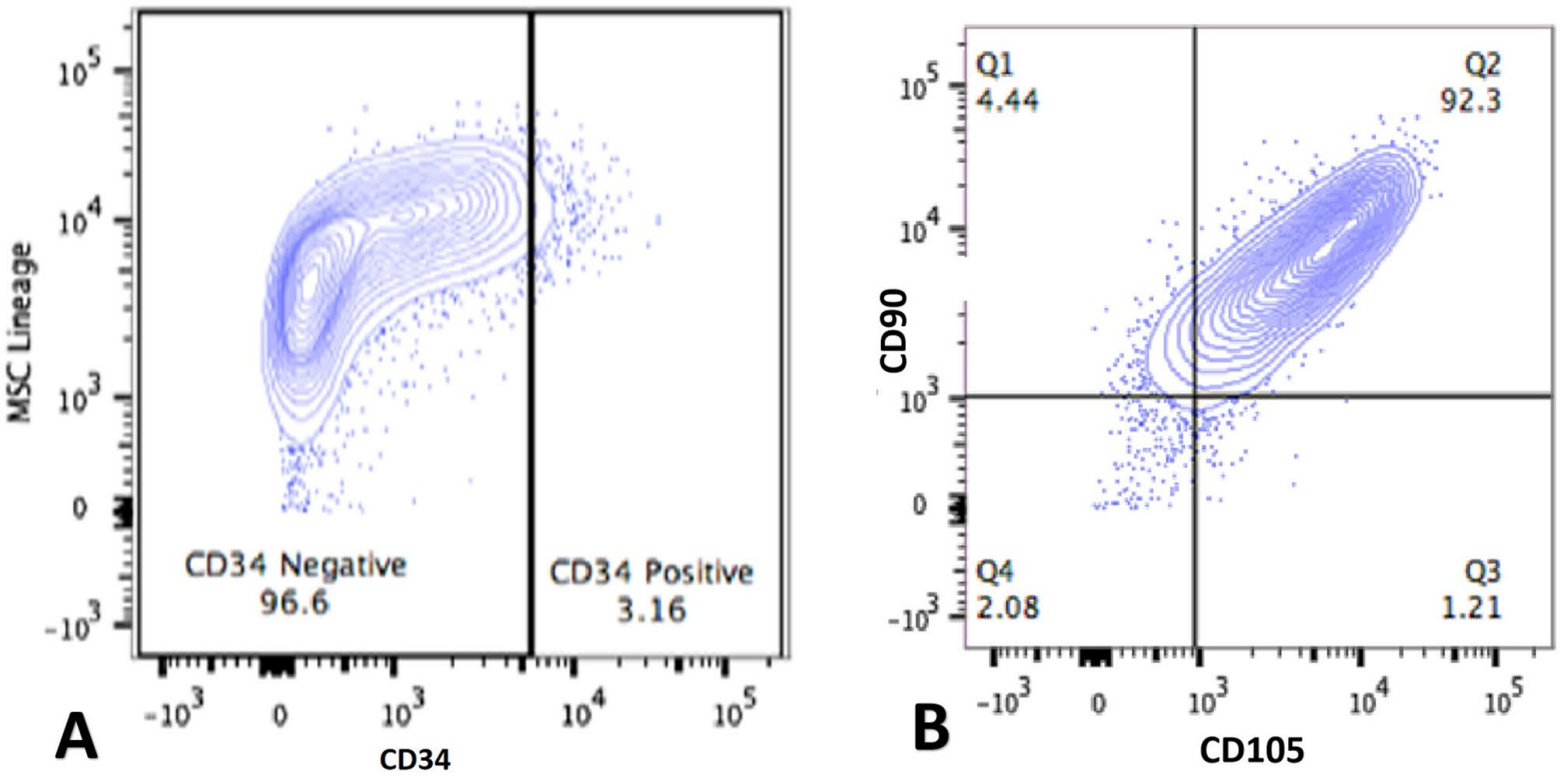
Flow Cytometry Analysis. (**A**) shows the negative expression of CD34 (hematopoietic stem cell marker) on WJ-MSCs lineage, (**B**) shows two parameters plots for WJ-MSCs labeled with CD90 and CD105, where Q1 denotes cells positive for CD90, Q2 double-positive cells, Q3 positive cells for CD105, and Q4 negative cells for both markers.

**Fig. 3:**
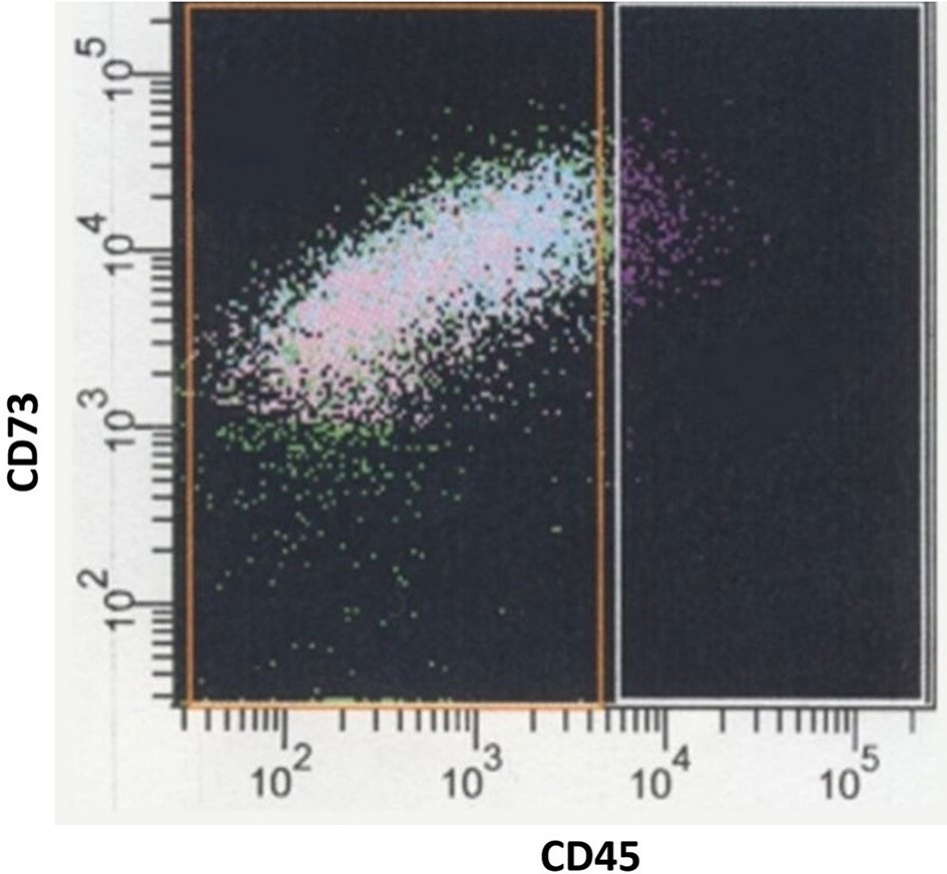
Flow Cytometry Analysis showing the negative expression of CD45 and the positive expression of CD73 (hematopoietic stem cell marker) on WJ-MSCs lineage.

Using the International Society for Hematotherapy and Graft Engineering (ISHAGE) procedure^19^, flow cytometric analysis was used to determine if the isolated cells were multipotent WJ-MSCs and to evaluate the WJ-MSCs yield. The BD Accuri TM C6 Cytometer (Becton, Dickinson and Company) was used. The program System (Becton, Dickinson and Company) was used for analyzing the data^20^. According to^21^, the WJ-MSCs were characterized by positivity of CD73, CD90, CD105 and negativity of CD34 and CD45 on flow cytometry analysis as shown in Figs. 2, 3.

In brief, after being re-suspended in 1 ml of PBS including 1% FBS, the cultured WJ-MSCs were labeled with 5 μl of fluorescein isothiocyanate (FITC), APC anti-human, and phycoerythrin (PE)-conjugated antibodies as positive markers for CD105, CD90, and CD73 respectively. Antibodies conjugated with phycoerythrin cyanine 5 (PE-Cy5) and PE-Cy7 were utilized as negative markers for CD45 and CD34, respectively. As controls, FITC, PE, and PC5 matched isotype antibodies were used. All antibodies were from BD Biosciences except CD90,and CD73 were from Miltenyil Biotec. At 4 °C and in the dark for 45 min, antibodies were added at a concentration of 1.5 µl in 50 µl of cell suspension for CD73 and CD90 and 1 µl in 50 µl of cell suspension for CD105, CD34, and CD45 as recommended by the manufacturer, then the yield was assessed using flow cytometry.

### Statistical analysis

The collected data was revised, coded, tabulated and introduced to Statistical package for Social Science (IBM SPSS Statistics, Version 25.0. Armonk, NY). Mann Whitney-U or student t-test was used for comparing numerical variables between groups. Chi-square test was utilized to compare categorical variables. Correlation analysis was used for studying the strength of correlation between two numerical variables. The correlation between WJ-MSCs yield at the end of P3 and P1 doubling time and the potential numerical maternal and newborn predictors was tested by Spearman correlation coefficient. A correlation coefficient of less than 0.3, between 0.3 and 0.7 and of more than 0.7 indicated weak, moderate and strong linear correlations, respectively.

## Results

### Demographics

At the Mansoura University Hospital delivery unit, UC samples were obtained from 100 singleton newborns who were born via Caesarean section. Twenty-one specimens were excluded because, in 14 samples, the material was contaminated, and in 7 samples, there was no apparent cellular growth after 20 days. The final analysis had 79 specimens in total. The maternal data were as follows: the mean parity was 1.2 ± 1.1, the mean gravidity was 2.4 ± 1.3, and the mean age was 29.3 ± 5.4 years, with a range of 18–42 years. Four (5.1%) of the mothers in the study have gestational diabetes; two were managed with diet, and the other four required insulin therapy. There were six mothers (7.6%) with pre-eclampsia. Of the 79 babies who were part of the study, 51 (64.6%) were female and 28 (35.4%) were male. Of the infants born between 26 and 41 weeks, 56 (70.9%) were full term and 23 (29.5%) were preterm. The mean gestational age was (36.3 ± 3.3). The median birth weight was 2.8 (1.3–4) kg, with a mean of 2.73 ± 0.6 kg. The median birth length was 45 (39–52) cm, with a mean of 45.5 ± 2.99 cm. Within the 30–35 range, the average newborn head circumference was 33.1 ± 4.8. Ten (12.7%) newborns had amniotic fluid stained with meconium, while sixty (75.9%) babies were cephalic presentation.

### Effects of neonatal and maternal variables on the passage (P1) doubling time

**Fig. 4:**
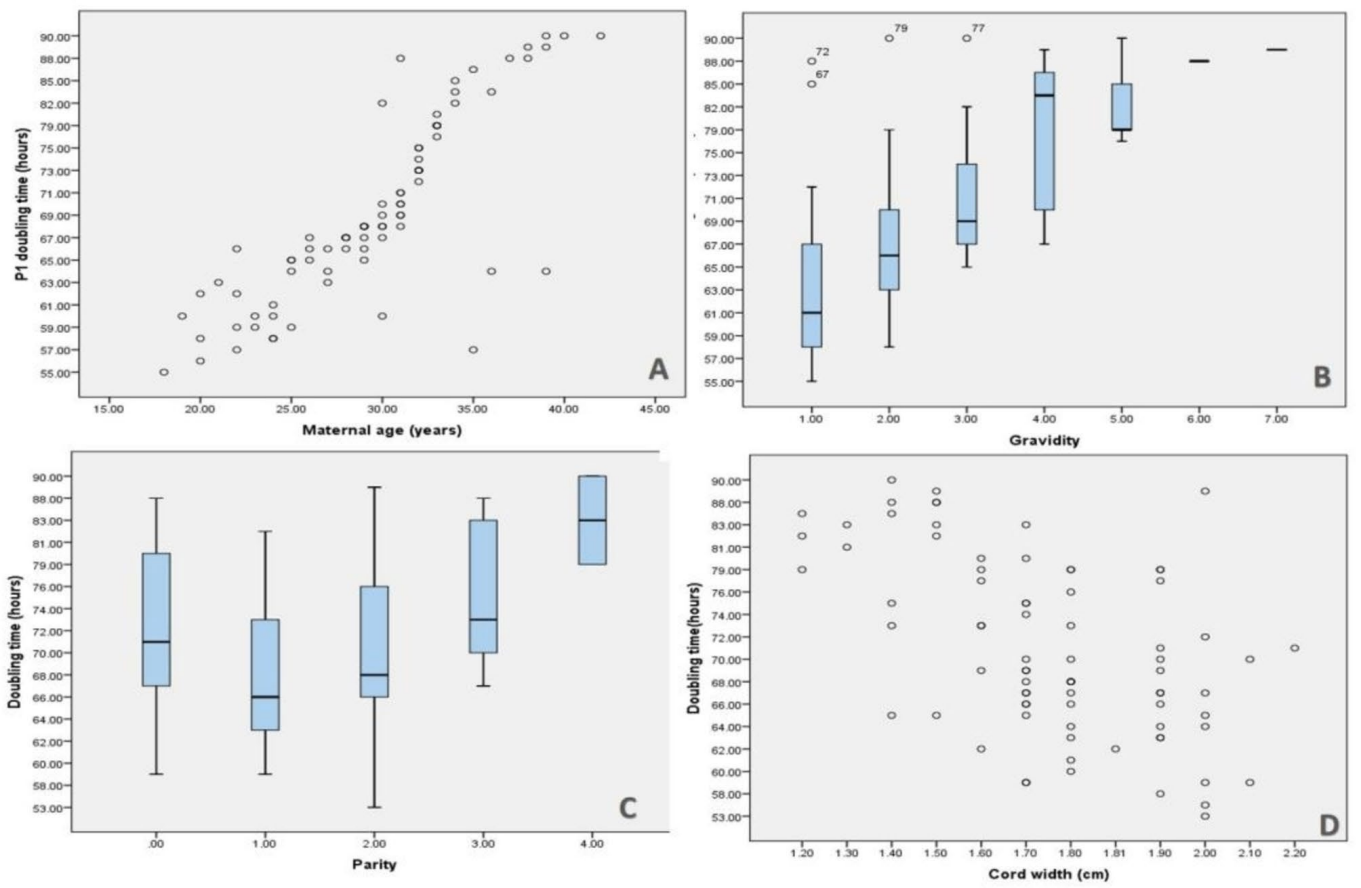
P1 doubling time and correlation between variables. (**A**) shows a scatter plot demonstrating the correlation between WJ-MSCs P1 maternal age and doubling time (*P* < 0.001, r = .827,), (**B**) a box plot demonstrating the correlation between P1 doubling time and gravidity (*P* < 0.001, r = 0.624), (**C**) a box plot demonstrating the relationship between P1 doubling time and parity (*P* < 0.001, r = 0.551), and (**D**) shows a scatter plot showing the correlation between umbilical cord width and P1 doubling time.

For WJ-MSCs, the average P1 doubling time was 68 (55–90) h. When the association between P1 doubling time and maternal variables were analyzed, Fig. 4A–C showed that P1 doubling time had a small positive correlation with gravidity (r = 0.624, *P* < 0.001), parity (r = 0.551, *P* < 0.001), and a strong positive correlation with maternal age (r = 0.827, *P* < 0.001). P1 doubling time was not significantly associated with any of the neonatal variables: birth head circumference (*P* = 0.543), birth length (*P* = 0.236), birth weight (*P* = 0.735), or gestational age (*P* = 0.186). There was no statistically significant difference in the median doubling time between preterm newborns (69 h) and full term babies (67.5 h) (*P* = 0.612). Additionally, there was no statistically significant difference in the hours of male (66) and female (68) babies (*P* = 0.612). The presence of amniotic fluid stained with meconium (*P* = 0.238) or fetal mal-presentation (*P* = 0.502) had no discernible effect on the P1 doubling time. Double time and UC width were shown to have a statistically significant negative connection (r = −0.5, *P *= 0.001) (Fig. 4D).

### Maternal and neonatal variables' effects on the yield of WJ-MSCs

By the end of P3, the mean WJ-MSCs yield was (2.34 ± 0.74 × 10^6^) cell/ml [2.6 (0.4 – 3.5) × 10^6^ cell/ml], according to the median (range). For the median (range), the viability percentage was 83.1 ± 7.6% [84.5 (65–97)%]. UC samples were used to determine the WJ-MSCs count of full-term neonates (2.37 ± 0.74 × 10^6^ cell/ml; median = 2.6 (0.4–3.5) × 10^6^ cell/ml) and preterm newborns (2.27 ± 0.76 × 10^6^ cell/ml; median = 2.6 (0.7–3.3) × 10^6^ cell/ml). No statistically significant difference was seen in the median WJ-MSCs yield between preterm and full-term newborns (Mann–Whitney Up = 0.742).

Maternal age and WJ-MSCs yield had a negative correlation (r = -0.246, *P* = 0.029) according to an analysis of maternal variables (Table 1). There was no statistically significant difference in the median WJ-MSCs yield between UC specimens obtained from women with and without gestational diabetes. In UC specimens collected from mothers with or without pre-eclampsia, WJ-MSCs yield was also similar. It is notable, therefore, that there were a very few pre-eclamptic or gestational diabetic individuals in the study. WJ-MSCs yield did not significantly correlate with maternal hemoglobin level prior to birth, parity, or gravidity.

**Table 1:** A summary of the correlation between neonatal and maternal variables and the yield of WJ-MSCs.

Maternal variables	Correlation coefficient	*P* value
Mother's age (years)	−.246*	0.029*
Mother's hemoglobin level (gm/dl)	0.037	0.743
Parity	0.069	0.545
Gravidity	0.003	0.982
Toxemia		0.732**
Gestational diabetes		0.762**
Neonatal variables		
Gestational age in weeks	0.329	0.004*
Sex		0.681**
Fetal presentation		0.373**
Newborn birth weight (kg)	0.272	0.015*
The width of Umbilical Cord (cm)	0.285	0.011*
Birth length (cm)	0.057	0.620
The head circumference (cm)	0.130	0.253
Meconium stained amniotic fluid		0.308**

*P values < 0.05, **Mann–Whitney U test.

Analyses of the association between WJ-MSCs yield and newborn characteristics (Table 1) revealed a positive correlation between the WJ-MSCs count and birth weight and gestational age. However, it was shown that the yield of WJ-MSCs and UC width had a weakly positive correlation. A multivariate linear regression analysis revealed that the sole independent predictor of WJ-MSCs yield was maternal age (Table 2). The yield of WJ-MSCs was not independently predicted by gestational age, birth weight, or cord breadth (*P* = 0.330).

**Table 2:** The WJ-MSCs yield model using linear regression analysis.

Variables	Standardized ẞ	*P*-value
The age of gestation	−0.151	0.563
Maternal age	−0.249	0.027
Neonate birth weight	0.302	0.051
The width of Umbilical Cord	0.210	0.330

(Adjusted *P* = .003, R^2^ = .146).

## Discussion

A promising source of MSCs for therapeutic uses is Wharton's Jelly. WJ-MSCs are easily accessible with many advantages over the BM-MSCs. Several maternal and neonatal parameters have been studied in relation to the characterization and quality of hematopoietic UC blood stem cells in the past. Although research on how maternal and neonatal variables affect the yield of WJ-MSCs is still under research investigation. For the purpose of aiding in finding and selecting the ideal UC donors, this study studied the effects of many maternal and fetal factors on the yield of WJ-MSCs.

According to our findings, the mother's age has a substantial impact on the yield of WJ-MSCs. This is in agreement with the findings of^22^, who found that whereas UC-MSCs from elder donors show diminished differentiation potential, UC from younger donors is a reasonably abundant source of MSCs. Furthermore, compared to younger age groups, there was a significant negative correlation in the expression levels of both CD29 and CD105, according to^23^. It was reported that as a woman ages increase, her mesenchymal stem cells expressing the BIRC2 and BIRC3 genes decrease as stem cells obtained from younger women may exhibit more apoptotic resistance and a more stem cell-like quality, which may enhance their therapeutic potential and clinical usability^16^.

A known pluripotent transcription factor, SOX2 is implicated in reprogramming, self-renewal, and maintaining the homeostasis of stem cells. The SOX2 gene is statistically considerably more expressed in WJSC in women under the age of 34 than it is in women over 34, according to^24^. Furthermore, a statistically significant moderate negative correlation is demonstrated between the SOX2 gene expression and the maternal age. This result was clarified by^25^ and^26^, who found that aging in mothers is related to telomere shortening and increased susceptibility to apoptosis, and that stem cells' telomere length could not be maintained by the level of telomerase activity.

These results largely stated the decline in CD105, CD73, and CD90 expression levels that they reported in their study's UC-MSCs as they were older. Furthermore, it has been previously reported how aging affects adult stem cells^27^ found a decrease in adult MSCs number derived from the bone marrow with aging. According to^28^, there is a decrease in the quantity, functionality, and multi-lineage differentiation characteristics of mature MSCs as they age. Additionally, the authors hypothesized that the lower differentiation capacity of the older donor's umbilical cord cells could be related to the declining functional state of the mother's older organs, which support and foster the development of umbilical cord MSC^22^. Conversely to this finding, maternal age had no effect on the quality of WJ-MSCs, according to^29^.

As per the study carried out by^30^, vaginal and cesarean births had MSC cell yields that were comparable. According to Penolazzi et al.^29^, the method of delivery had no impact on the characteristics or survivability of MSCs. To reduce the incidence of contamination, we only used specimens from Cesarean births in our investigation. We observed that the WJ-MSCs count and doubling time were not more impacted by birth order. A reduction in the number of WJ-MSCs was correlated with increasing parity, nevertheless the relationship was not statistically significant. Birth order and viability showed a moderately positive association (r = 0.119, *P* = 0.044), whereas parity and the amount of UC blood showed no correlation (*P* = 0.057)^31^. According to^32^, WJ-MSCs obtained from younger women who gave vaginal delivery have increased expression of the BIRC2, BIRC3, and BIRC5 genes.

Pre-eclampsia had no obvious effect on the population doubling time or WJ-MSC yield in this study. In contrast, pre-eclampsia has been shown by^33^ to result in a considerable increase in cell count and reduction in the fraction of immature cells and increasing the mature cells. Compared to normal samples, the proliferation rate of early-passage gestational diabetes mellitus (DM) samples was significantly lower^34^ . Furthermore, with a mean cell density of 1.9 × 10^4^ cells per plate in gestational DM samples as opposed to 4.1 × 10^4^ cells per plate in normal samples, the population doubling rate was considerably lower in these samples after 12 days of incubation. Gestational DM-UC-MSCs did not grow above passages 6 or 7. All UC-MSCs had comparable amounts of the stem cell markers CD90, CD73, and CD105 during early stages. These results are consistent with a study conducted by^35^, which found that MSCs derived from Wharton Jelly of the DM group grew and proliferated at a much slower rate in all passages than MSCs isolated from a control group. The quantity of non-viable cells in the gestational DM samples significantly increased than viable cells.

The WJ-MSCs count was shown to be statistically significantly impacted by gestational age in our investigation, although there was not a significant association established between gestational age and P1 doubling time. Nevertheless, there was no statistically significant difference in the median MSC output between full-term and preterm newborn when comparing them to full-term babies. Fourty five infants were divided into three groups in a study conducted by^36^ according to their gestational ages: group A (28–31 weeks), group B (32–35 weeks), and group (C) ≥ 36 weeks. Even after adjusting the birth weight, maternal co-morbidities, and prenatal steroid use, the authors still found a significant inverse correlation between gestational age and MSC yield. Similar findings were reported by^37^, who found that despite both samples maintained a high rate of proliferation, preterm cord samples developed more MSC in all passages than term cords. On the other hand, according to^38^, the quantity of umbilical cord vein UC-MSCs from both full term and preterm patients is comparable, and both groups exhibit a high rate of proliferation. Penolazzi et al.^29^, on the other hand, found no correlation between gestational age and MSC yield.

Age at gestation may possibly have an impact on the UC-MSCs' potential to modulate immunity. According to^38^, preterm UC-MSCs do not have the same potent inhibitory effects on immune cell proliferation as full-term UC-MSCs. A lymphocyte co-culture experiment revealed that both full term and preterm UC-MSCs reduced peripheral blood mononuclear cell proliferation in the mixed lymphocyte response. Compared to preterm UC-MSCs, the term UC-MSCs had a stronger proliferation inhibition. The scientists explained the inhibitory difference by the preterm UC-MSCs' immature immune systems. The source, donor age, and passage number of cells can also have an impact on the immuno-modulatory qualities of MSCs^39^ and^40^.

According to^41^, placental weight, birth weight, and full term birth were the primary factors influencing cell proliferation. Despite the strong correlation between these parameters and gestational age, multivariate regression analysis revealed that gestational age was the only independent predictor of population doubling time (*P* = 0.0094). Our study found that the baby's birth weight had a substantial impact on the number of WJ-MSCs; a greater birth weight leads to increased the yield of MSCs. According to^30^, the yield of MSCs was not significantly affected by the mother's age or gestational age, but a greater birth weight was associated with a more yield, which is consistent with our findings. On the other hand, Gil-Kulik et al.^24^ found that lower birth weight correlates to higher SOX2 gene expression in WJ-SCs. SOX2 is a known pluripotent transcription factor that contributes to cell reprogramming and self-renewal. According to Penolazzi et al.^29^, the MSCs output was not significantly impacted by birth weight. The author explained that their study focused on the quality of the stem cells not the number so the study finding differs from other studies that correlate the number of isolated stem cells to the birth weight^31,42^.

According to our findings, there was no statistically significant difference in the WJ-MSCs yields in regard to fetal gender, presentation, length at birth, or head circumference. According to^36^, the mean MSCs number of female neonates was greater than that of male infants. When comparing MSCs separated from WJ from female individuals to MSCs obtained from WJ from male subjects, Balzano et al.^43^ observed that the expression of OCT4 and DNMT1 genes was considerably greater in WJ-MSCs derived from male participants. They came to the conclusion that gender significantly affects stemness genes. However,^29^ found that gender did not affect the quality of WJ-MSCs.

We discovered a statistically significant negative association between doubling time and UC width and a slight positive correlation between MSCs yield and UC width.

We discovered that the amount of meconium in the amniotic fluid had no effect on the production of MSCs or the population doubling time. According to^44^, there was little change in the expression of the mesenchymal markers CD90 and CD105 following exposure to meconium. Amniotic fluid MSCs may change in phenotype after being exposed to meconium, although they are still capable of differentiating.

To sum up, Wharton's jelly MSCs are a perfect and exciting source of MSCs. Numerous factors related to mothers and newborns may impact the MSC output and should be investigated for settling the optimal selection criteria for the ideal donors. Our research showed that a higher yield of MSCs produced from Wharton's jelly correlates to a younger mother's age, increased birth weight, and longer gestational age.

## Data Availability

The data that support the findings of this study are available on request from the corresponding author.
